# Research progress of metabolomics in cervical cancer

**DOI:** 10.1186/s40001-023-01490-z

**Published:** 2023-12-13

**Authors:** Yuhan Jia, Kun Zou, Lijuan Zou

**Affiliations:** 1 Department of Radiotherapy, The Second Hospital of Dalian Medical University, Dalian, Liaoning Province China; 2 Department of Radiotherapy, The First Hospital of Dalian Medical University, Dalian, Liaoning Province China

**Keywords:** Cervical cancer, Metabolomics, Metabolic regulation, Diagnosis, Treatment

## Abstract

**Introduction:**

Cervical cancer threatens women's health seriously. In recent years, the incidence of cervical cancer is on the rise, and the age of onset tends to be younger. Prevention, early diagnosis and specific treatment have become the main means to change the prognosis of cervical cancer patients. Metabolomics research can directly reflect the changes of biochemical processes and microenvironment in the body, which can provide a comprehensive understanding of the changes of metabolites in the process of disease occurrence and development, and provide new ways for the prevention and diagnosis of diseases.

**Objectives:**

The aim of this study is to review the metabolic changes in cervical cancer and the application of metabolomics in the diagnosis and treatment.

**Methods:**

PubMed, Web of Science, Embase and Scopus electronic databases were systematically searched for relevant studies published up to 2022.

**Results:**

With the emergence of metabolomics, metabolic regulation and cancer research are further becoming a focus of attention. By directly reflecting the changes in the microenvironment of the body, metabolomics research can provide a comprehensive understanding of the patterns of metabolites in the occurrence and development of diseases, thus providing new ideas for disease prevention and diagnosis.

**Conclusion:**

With the continuous, in-depth research on metabolomics research technology, it will bring more benefits in the screening, diagnosis and treatment of cervical cancer with its advantages of holistic and dynamic nature.

## Introduction

Cervical cancer poses a serious health risk to women because it has a precancerous stage that can last for years and can be reversed [1]. A number of studies have shown that 80–90% of 5-year cure rates are due to early diagnosis and treatment of cervical cancer patients [2]. Therefore, effective screening methods for cervical cancer have become an important tool for detecting, interrupting and reducing the occurrence of cervical cancer. However, the study shows that only 81% of women aged 21–65 are up-to-date on screening [3]. Currently, cervical cancer screening is mainly performed by cervical cytology, colposcopy, cervical biopsy, and high-risk Human Papillomavirus (HPV) [4], that often causes delays in many patients due to problems in obtaining the material. While the specimens used for metabolomic analysis are mainly biological fluids such as serum, plasma, and urine, which are rich in metabolic information, simple to obtain, and more acceptable to patients.

Metabolomics is a discipline proposed by Nicholson [5] in 1999 for studying the "mapping" of the metabolome from a holistic perspective. Metabolomics is a new field developed after genomics and proteomics [6], which focuses on the dynamic changes in the quality and quantity of endogenous metabolites (such as amino acids, fatty acids, sugars, vitamins and lipids) in living organism caused by external stimulation [7]. As serious metabolic disorders are common in tumor patients, scientists have obtained characteristic differential metabolic profiles and identified specific biomarkers through metabolomic studies of cervical cancer patients [8], which provide new ideas for research related to detection, screening, early diagnosis and treatment of cervical cancer.

## Metabolic changes in cervical cancer

Tumor tissue requires adequate nutrients and an energy supply during growth and proliferation. In adaptation to tumorigenesis and progression, there are significant changes in many aspects of tumor cell metabolism, such as glycolysis, tricarboxylic acid cycle, oxidative phosphorylation, amino acid metabolism, fatty acid metabolism and nucleic acid metabolism, which is called metabolic reprogramming of tumor cells [9]. Differential metabolite analysis of cervical cancer, cervical intraepithelial neoplasia and control tissues showed higher levels of isoleucine, leucine, valine, glycoprotein, methionine and lower levels of α-glucose and β-glucose in patients with cervical intraepithelial neoplasia compared to normal controls; patients with cervical squamous cell carcinoma had higher levels of lactate, alanine, isoleucine, methylhistidine acid, lipid low density lipoprotein (LDL) was higher and tyrosine, phenylalanine, α-glucose, β-glucose, creatine, shark inositol, and acetic acid were lower. Lactate, alanine, glycine, and LDL were higher in patients with cervical squamous cell carcinoma compared with patients with cervical intraepithelial neoplasia [10]; tyrosine, α-glucose, β-glucose, acetic acid, and phenylalanine were lower [8].

### Glucose metabolism

With the growth of tumor cells, the demand for glucose gradually increases, the rate of glycogenolysis and gluconeogenesis accelerates and the rate of glycogen synthesis decreases, resulting in a decrease in the content of glucose in tumor tissue [11]. In a study by Sitter [12], the content of α-glucose and β-glucose in cervical tissue was reduced in patients with cervical intraepithelial neoplasia and cervical cancer compared with healthy individuals, indicating that there are serious disorders of glucose metabolism in cervical cancer patients [13]. It is called Warburg effect that in the process of growth and proliferation, malignant tumors requiring large and rapid energy supply are quickly energized by their own anaerobic enzymes, which are converted to lactic acid by the action of lactate dehydrogenase, even if oxygen supply is sufficient [14]. The massive production and accumulation of lactic acid caused local acidosis in the tumor tissue. Acidosis leads to increased extracellular matrix breakdown and also promotes angiogenesis, which can directly lead to the metastasis of malignant tumor cells [15]. As a result, malignant tumor cells have more obvious proliferative advantages after surviving the acidic environment selection. Those with high lactate concentration in malignant tumor tissues are prone to metastasis and have a worse prognosis compared to those with low lactate concentration, which may be related to radiation resistance caused by hypoxia [16]. Previous HRMAS studies have shown that increased lactate content in cervical cancer tissues suggests increased anaerobic enzymes in cervical cancer patients [17]. Thus, increased glycolytic metabolism is an important marker for the existence of commonality in pathogenesis.

### Amino acid metabolism

The growth and proliferation of tumor tissues require large amounts of amino acids to support their exuberant protein and nucleic acid synthesis and energy metabolism [18], so there is a specific demand for certain amino acids. Compared to healthy controls and cervical intraepithelial neoplasia, cervical cancer has elevated levels of the glycogenic amino acids alanine and glycine; in contrast, phenylalanine and tyrosine are decreased [19]. As an important participant and regulator of glucose metabolism, alanine is an intermediate product of glucose and amino acid metabolism. There are two main pathways of its metabolism in the body: glutamine and other amino acids generate alanine by the action of transaminases; glucose is glycolysed to produce pyruvate, which combines with ammonia to generate alanine [20]. Under certain conditions, alanine can be deaminated by alanine aminotransferase to produce pyruvate. It is the production of pyruvate by glycolysis that is associated with cervical carcinogenesis, which increases the level of alanine [21]. Therefore, the elevated alanine content in cervical cancer tissues may be the result of a combination of sugar and amino acid catabolism.

Glycine provides methyl groups in the synthesis of proteins and nucleotides and is an important raw material for the synthesis of purine nucleotides. The rise in glycine content in cervical cancer tissues is both due to increased glycolysis metabolism and required for protein and nucleic acid biosynthesis [22].

Phenylalanine is one of the essential amino acids in the body and is converted into tyrosine by the action of alanine hydroxylase [23]. Tyrosine and phenylalanine are involved in sugar and fatty acid metabolism in the body and at the same time affect the levels of neurotransmitters and hormones in the body. In previous studies, the levels of tyrosine and phenylalanine decreased more significantly in colorectal cancer [24], so they can be used as one of the more reliable diagnostic indicators. The simultaneous decrease of both of them in cervical cancer tissues might be the result of the mutual influence and regulation of both metabolic pathways.

It was reported that some amino acid levels were higher in cancer tissues than in normal controls [25], and several amino acids were significantly increased both in cervical cancer tissues and in blood of cervical cancer patients [26]. The metabolic levels of L-O-phosphoserine and L-asparagine were also found to be increased in cervical cancer tissue samples, which may be related to glycolysis.

Glutamate (Glu) is reduced in cervical cancer tissues. In the blood, about 25% of glutamate is present in the form of glutamine [27]. Glutamine (Gln) is the source of nitrogen for the synthesis of other amino acids and proteins and is the main energy provider for rapidly growing cells such as fibroblasts, lymphocytes and intestinal mucosal cells [28]. Recently, it has been found that the source of nutrients for many tumors has shifted from glucose to Gln, which is able to provide nitrogen and carbon to tumors to promote their growth [29]. Some in vitro studies have found that tumor cell growth is dependent on glutamine [30]. The consumption of glutamine by tumors is in a form similar to the Warburg effect, characterized by its rapidity and inefficiency. Accordingly, it has been proposed that abnormal glutamine catabolism could be another feature of tumor metabolism [31]. The low levels of glutamine in cervical cancer tissues may be due to the need for sufficient energy in the growth of cancer cells. Glutamine metabolism is probably an additional energy replacement pathway in cancer cells [32], and the abrupt increase in glutamine metabolism consequently results in a reduction in its level.

### Lipid metabolism

Lipids are significantly higher in cervical cancer tissues. The LDL receptors on the surface of malignant tumor cells are very sensitive to cholesterol, as well as with a substantial increase in the demand for cholesterol by malignant tumor tissues, which results in elevating the levels of LDL that carry cholesterol [33]. This study [34] is the first to identify C8-ceramide-1-phosphate as an important lipid metabolite that modulates cervical cancer cell function. In addition, they also identified that other lipids, such as LysoPC and PC, may also involve cervical cancer progression, requiring further investigation. It was found in a study [35] based on HRMAS1HNMR technique that lipids were found to be significantly elevated in cervical cancer tissues. Furthermore, lipids have an important regulatory role in the transport and secretion of oncogene proteins, and those involved in post-transcriptional modifications all have an extremely important impact on tumorigenesis development [36]. Thus, the inhibition of lipid synapse as a target for the treatment of tumors is promising for important research.

In the body, creatine is synthesized with glycine as the backbone and then transformed into phosphocreatine, the high-energy phosphate bond that stores ATP, an important compound for energy storage and utilization [37]. The decrease in creatine content has shown that the body is in a state of prolonged energy depletion and there is a disruption of energy storage and energy metabolism in the body [38]. It also indicates that the decrease in creatine and inositol content is associated with enhanced fat mobilization [39]. This may be related to the depletion of energy metabolism in cervical cancer patients.

### Nucleotide metabolism

There are significantly higher levels of 5'-inosinic acid, uridine 5-monophosphate, uridine 5'-triphosphate, and 5'-adenine nucleotide in cervical intraepithelial neoplasia compared to normal cervical epithelial cells [40], which are involved in purine and pyrimidine metabolism, suggesting that cell proliferation may accelerate the tumor formation process. Altered metabolism of pyrimidine and purine, as substrates and nitrogen sources, may be associated with a higher rate of tumor cell multiplication [41]. Compared to cervical intraepithelial neoplasia, cervical cancer tissues have higher levels of 3'-methoxyguanosine, histamine, phenyllactic acid, citraconic acid, guanosine, cholesterol sulfate, etc. [42].

### Purine metabolism

During the study, the urine of cervical cancer patients and healthy individuals was tested by UHPLC–MS method, and 17 potential biomarkers and trends were identified by statistical calculations, and 12 compounds [Table 1], including uric acid, were identified as possibly related to the pathogenesis and metabolic pathways of cervical cancer tumors [43]. The determination of uric acid is important in cervical cancer [44] and in the syndrome of dysregulated antidiuretic hormone secretion, and metabolic disorders can also lead to an increase in uric acid. Some recent studies have shown that changes in uric acid levels reveal the process of cervical cancer development and that blood uric acid concentration is highly correlated with the degree of cervical cancer development and is a risk factor for inflammatory-like diseases [45]. Uric acid is an intermediate in the conversion of glutamate to histidine. Downregulation of uric acid may be associated with impaired histidine metabolism in tumor tissues or disorders of glutamate metabolism. Ogihara [46] showed that uric acid and its oxidation products can be used as a marker of free radical generation in the body.

**Table 1:** Identified potential biomarker compounds in urine of CC patient in the study of Lu Jin [47]

No.	Compound	Trend
1	Pyridylacetic acid	Up
2	Tyrosine	Up
3	3-Methylxanthine	Up
4	Betaine	Down
5	Mannitol	Up
6	Uric acid	Up
7	Proline betaine	Down
8	Amino malaric acid	Up
9	1-Methylguanine	Up
10	Theobromine	Up
11	Tryptophan	Up
12	Theophylline	Up

Betaine provides methyl and amino acids [48], which are involved in the processes of amino acid metabolism, lipid metabolism and DNA methylation in the body. Low concentrations of betaine in urine can trigger disorders of lipid metabolism and related inflammation. The variation of mannitol and aminomaluronic acid levels in urine is also a metabolic marker reflecting the inflammation of the body [49]. The above detectable metabolites may be independent risk factors for the development of cervical cancer.

### Choline metabolism

Glycerophosphoryl choline is increased in cervical cancer. Glycerophosphoryl choline as choline derivative, choline is one of the components of cell membrane phospholipid metabolism [50], which is involved in the synthesis and degradation of cell membrane and acts as an important role in the metabolic process of tumor cells [51]. The content of free-choline is at very low level in normal living tissues, while the content is elevated in malignant tumors, which can reflect the transport of tumor cell membranes and cell proliferation, and the content of choline correlates with the benign and malignant degree of tumors. An increased level of choline was found in the plasma of the cervical cancer patients [26]. Choline has three main metabolic pathways in organisms: phosphorylation, oxidation and acetylation, and the one most closely related to tumors is the choline phosphorylation pathway [52]. During the malignant transformation of tumor cells, choline kinases are activated, leading to increased levels of phosphorylcholine. The tumor cells with accelerated proliferation contain large amounts of phospholipids, especially lecithin, which, as an important component of the cell membrane structure, lecithin and other lipoproteins can also regulate cellular information transduction processes [51], thus affecting cell proliferation and differentiation.

There are higher levels of 3′-methoxyguanosine, histamine, phenyllactic acid, citraconic acid, guanosine, cholesterol sulfate, β-D-glucosamine, L-2-aminoadipic acid, etc., in cervical cancer tissues compared to CIN [53]. Cervical cancer tissues have obvious metabolic complexity involving a large number of metabolites and metabolic pathways, and the diagnostic aid of a single metabolite is limited. Therefore, it is difficult to provide one metabolic biomarker of sufficiently high diagnostic value. However, a combination of more metabolic analytes can provide a comprehensive view of the pathological state of cervical cancer.

### Metabolic pathways

Taurine and taurine metabolic pathways can be assumed that this pathway is closely related to the pathological process of cervical cancer and many evidences suggest that it is one of the key pathways of tumor progression [54]. Taurine is a sulfur-containing amino acid that is converted by dehydrogenation of the enzyme taurine dehydrogenase [55]. Taurine is connected to alanine and glutamate metabolism and acts as a critical role in osmoregulation, antioxidant, detoxification and stimulation of glycolysis and gluconeogenesis [56]. A study on breast cancer reported that the potency of taurine was clearly reduced in the serum of breast cancer patients compared to high-risk patients and healthy controls, indicating the importance of taurine for the early warning of malignant transition in the breast [57]. The presence of taurine in the urine of bladder cancer patients may be a biomarker for bladder cancer detection [58]. Taurine is raised in colon cancer [59], so taurine is important for the prevention and avoidance of progression of cervical carcinogenesis.

**Fig. 1:**
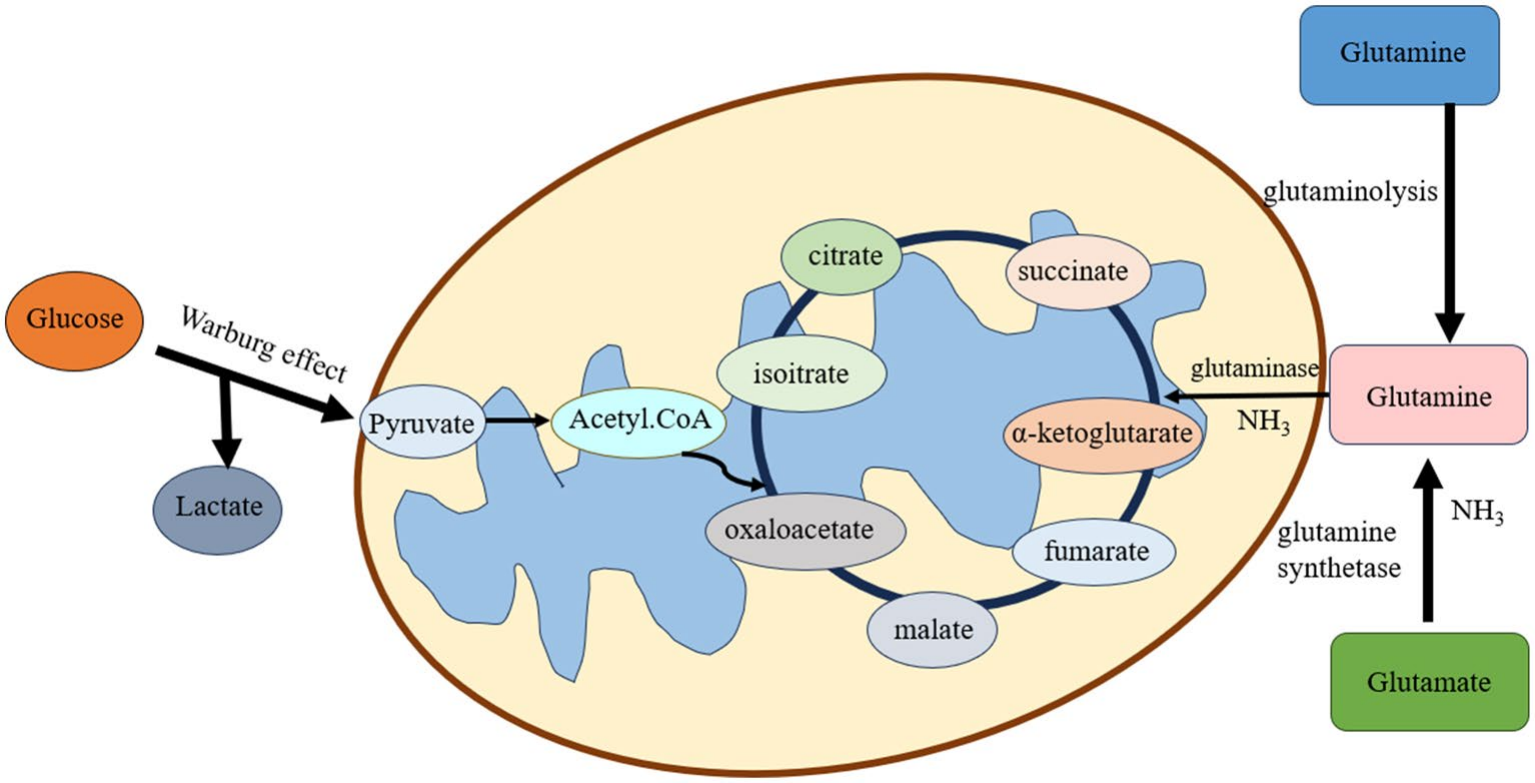
Glutamine, glucose and glutamate are imported into the cytoplasm of a cell. Glucose is depicted to be converted primarily to lactate via aerobic glycolysis or the Warburg effect or channeled into the mitochondrion as pyruvate and converted to acetyl-CoA for oxidation. Glutamine is used for different processes, such as glutaminolysis, which involves the conversion of glutamine to glutamate and ammonia by glutaminase

Alanine, aspartate and glutamate metabolism and glutamine and glutamate metabolism are also important pathways associated with cervical carcinogenesis [60]. Tumor cell metabolic patterns show significant changes in order to adapt to tumorigenesis and progression, such as enhanced glutamine catabolism [61]. In addition to the Warburg effect, tumor cells supply energy metabolism through glutamine catabolism [Fig. 1]. As well as consuming glucose for energy supply, tumor cells also supply the energy and biomolecular raw materials required for their growth and proliferation through glutamine catabolism [62]. Glutamine is mainly derived from the metabolism of its precursor energy storage form, glutamine. The metabolism of glutamine provides not only ATP, but also precursors necessary for the growth of tumor cells [63].

In Ning’s study demonstrated that CCDC106 inhibits p53-mediated apoptosis, leading to the progression of breast and cervical cancer with wtp53, while its phosphorylation by CK2 is required for its interaction with p53 and oncogenic function [64]. This study identified a CK2/CCDC106/p53 signaling axis in cancer progression, which may represent a new therapeutic target for cancer treatment.

## Application of metabolomics in the diagnosis and treatment of cervical cancer

### Diagnosis

Cervical intraepithelial neoplasia (CIN) is a group of cervical lesions closely related to invasive carcinoma of the uterine cervix, and timely diagnosis and correct management of CIN can reduce the morbidity and mortality of cervical cancer [65]. Currently, histopathology and cytopathology are mainly used to diagnose CIN clinically, but histopathology is highly invasive and cytopathology has a high rate of false negatives and false positives [66]. Sitter [12] studied the tumor tissues of eight cervical cancer patients using high-resolution magic angle rotation combined with principal component analysis, and found that the content of choline and amino acid fragments in tumor tissues was high, while the content of glycans was low. In the study [67], they have demonstrated that automated LA-REIMS analysis of LBC-derived cell pellets can be used to distinguish women with or without a hrHPV infection with high diagnostic accuracy (94% sensitivity and 83% specificity). The emphasis of primary population-based screening is high sensitivity with low false negative rate in order not to miss disease, so this technique is expected to be a new tool for early diagnosis of CIN.

The differences in systemic metabolic response profiles between the tumor and non-tumor groups were mainly in 12 metabolites such as formic acid, β-glucose, glycine, carnitine and very low density lipoprotein, which can be used as potential biomarkers to identify cervical cancer [68].

### Treatment

Surgery is the main treatment for cervical cancer, adjuvant treatment mainly includes neoadjuvant chemotherapy, simultaneous radiotherapy, and intensity-modulated radiotherapy [69], which is the current research hotspot in tumor treatment and can reduce tumor size, give patients access to surgery, and increase radiotherapy sensitivity, while tumor cells have different sensitivity to chemotherapy drugs [70], therefore, a study collected a total of neoadjuvant chemotherapy-sensitive, partially sensitive, and completely insensitive plasma from 38 cervical cancer patients was collected. The LC–MS and multivariate data analysis revealed that levalerine and levotryptophan could be used as potential markers to predict the sensitivity of cervical cancer patients to neoadjuvant chemotherapy. The study [71] found that tumor patients who are insensitive to certain chemotherapeutic drugs should change their treatment regimen in time to avoid further disease development and achieve individualized treatment for tumor patients.

Chai [72] used stool specimens to analyze metabolites associated with radiotherapy-induced intestinal symptoms (RIAIS), a common complication of radiation therapy for cervical cancer [73], and then identified metabolite differences compared to their own pre-radiotherapy and non-RIAIS groups. This metabolic profile can be developed as a clinical tool for RIAIS diagnosis or treatment monitoring. The use of metabolomics techniques has led to numerous studies in the pharmacological treatment of cervical cancer, such as: altered copper transporter protein and glutathione metabolism in KB-8-5-11 cells [74], a model of drug-resistant cervical cells overexpressing P-glycoprotein, which contributes to their recognition of a cisplatin-sensitive phenotype [75]; and the antiviral drug lopinavir, which increases membrane transporter protein activity and increases drug sensitivity in HPV-infected cells [76].

Metabolomics can also be used for the evaluation of the efficacy of cervical cancer treatment. Lei Gong Tang is a traditional Chinese herbal medicine with abundant resources in China, and Lei Gong Tang lactone alcohol is one of the most representative active ingredients, which can inhibit the spread of prostate cancer cells [77] and has good antitumor activity. There are some studies to act Lei Gong Tang on isolated cervical cancer cells and evaluate its efficacy using metabolomic methods. In Hu's study [78], isolated cervical cancer cells after the action of Radix et Rhizoma were used as the experimental group, and isolated cervical cancer cells after the action of cisplatin, adriamycin hydrochloride and paclitaxel were used as the control group, and their cellular metabolites were collected and metabolites were detected by ion trap gas chromatography mass spectrometry. The study confirmed that Radix et Rhizoma had the effect of inhibiting the proliferation of cervical cancer cells and promoting their apoptosis, but its anticancer mechanism needs further study. Both small molecules caffeic acid and metformin are capable of disrupting energy homeostasis [79], regulating oxidative metabolism in cervical tumor cells in regard to specific metabolic phenotype of the cells. Caffeic acid and metformin may provide a promising approach in the prevention of cervical cancer progression.

## Conclusions

Cervical cancer is a complex disease with multifactorial and multisystemic functional flocculation that can lead to metabolomic changes and pathophysiological alterations of the disease. It has been found that biomarkers of intermediates and end products of abnormal metabolic processes can be used to study tumorigenesis and progression.

Nevertheless, despite significant advancements in metabolomics methods, the identification of metabolite biomarkers that can distinguish cancer patients from healthy controls and many novel findings on altered cancer metabolism, many challenges persist in developing the routine clinical utility of metabolomics for cancer diagnosis. In addition, a major challenge for metabolomics is the interference by contributions from confounding factors such as diet, age, gender, ethnicity, drugs, lifestyle and environment [80]. The sensitivity of metabolism to biological changes means that metabolite levels are affected not only by disease processes, but also by many others. With approximately 5000–8000 small molecule metabolites present in the human body (excluding lipids), the identities of most of these are still unknown or at least difficult to measure. Biological specimens used for study are extremely complex; no single analytical technique is currently capable of detecting all the metabolites in a single step. These challenges are in fact well recognized and there are already major efforts to overcome them. Therefore, this will bring greater difficulties to clinical work, for example, studies have shown that the metabolites between races are different [81]. Secondly, there are 5000–8000 metabolites in the human body, most of these are still unknown, which is one of the main reasons that the existing results cannot be directly applied to the clinical utility. The application of metabolite to the clinic requires more molecular theory support, and when we learn more about the pathway of metabolite production, we will be better able to integrate it with clinical cases. We think that we may not have found the specific numerical range of the changes of metabolites, but their change rules are discernable. According to the above experiments, the trend of these metabolites is similar.

By directly reflecting the changes in the microenvironment of the body, metabolomics research can provide a comprehensive understanding of the patterns of metabolites in the occurrence and development of diseases, thus providing new ideas for disease prevention and diagnosis. Metabolomics testing is less damaging and more acceptable to the patient than doing puncture for pathology. It can reduce the patient's fear and pain, which have a greater advantage in the initial screening, it will be able to detect asymptomatic early-stage patients earlier. In clinical work, pathologic diagnosis is time-consuming and often bring high monetary costs. However, metabolomics is often judged by blood or urine. The acquisition method is less damaging and less costly, and can quickly provide the basis for diagnostic and therapeutic programs.

Metabolomics has an advantage in the field of histological research by analyzing the physicochemical properties of endogenous small-molecule metabolites in the human body with a variety of analytical instruments. By analyzing the total metabolic components of the body and finding specific biomarkers from them, timely, accurate, highly sensitive and highly specific diagnosis of clinical diseases can be made. With the continuous in-depth research on metabolomics research technology, it will bring more benefits in the screening, diagnosis and treatment of cervical cancer with its advantages of holistic and dynamic nature. In future clinical trials we could conduct controlled trials in different populations of different ages, ethnicities, living regions and lifestyles to determine whether there is still variability in their metabolites in the absence of the effects of cancer disease. If there is variability across populations, this will affect the clinical significance of what they represent. Instead of prescribing a certain interval for determining the diagnostic significance of metabolites, we might consider determining the clinical significance of these metabolites by individual trends, which might eliminate the anomalies caused by errors in different populations. We need to do more research on the molecular structure, many machines have been able to analyze the structure of metabolites, but their origin and mechanism of action are still unclear. We can apply C13 and other tracking methods to track the metabolites in the metabolism of the pathway, in order to clarify the significance of its representative. Through the current experiments, we can know that there are differences in metabolite trends in different cancer diseases. In the future, we can select more specific metabolites by comparing the metabolite differences between different cancers. Their expression trends in different tumors will be studied more thoroughly, thus improving the specificity of metabolites in cancer diagnosis. The accuracy of metabolites in cancer diagnosis requires strict identification of other diseases associated with the production of such metabolites, which requires more basic experimental research. As more markers are discovered, the accuracy will be higher. We believe that metabolomics will make great progress in the diagnosis, treatment, and prognosis of cervical cancer with the advancement of various studies.

## Data Availability

Not applicable.

## Abbreviations

CIN: Cervical intraepithelial neoplasia
HPV: Human papillomavirus
Glu: Glutamate
Gln: Glutamine
LDL: Low density lipoprotein
RIAIS: Radiotherapy-induced intestinal symptoms
